# The Warburg Effect Mediator Pyruvate Kinase M2 Expression and Regulation in the Retina

**DOI:** 10.1038/srep37727

**Published:** 2016-11-24

**Authors:** Raju V. S. Rajala, Ammaji Rajala, Christopher Kooker, Yuhong Wang, Robert E. Anderson

**Affiliations:** 1 Department of Ophthalmology, University of Oklahoma Health Sciences Center, 608 Stanton L. Young Blvd, Oklahoma City, OK 73104, USA; 2 Department of Physiology, University of Oklahoma Health Sciences Center, 940 Stanton L. Young Blvd, Oklahoma City, OK 73104, USA; 3 Department of Cell Biology, University of Oklahoma Health Sciences Center, 940 Stanton L. Young Blvd, Oklahoma City, OK 73104, USA; 4 Dean McGee Eye Institute, 608 Stanton L. Young Blvd, Oklahoma City, OK 73104, USA; 5 Oklahoma Center for Neuroscience, 975 N.E. 10^th^ street, Oklahoma City, OK 73104,USA

## Abstract

The tumor form of pyruvate kinase M2 (PKM2) undergoes tyrosine phosphorylation and gives rise to the Warburg effect. The Warburg effect defines a pro-oncogenic metabolism switch such that cancer cells take up more glucose than normal tissue and favor incomplete oxidation of glucose, even in the presence of oxygen. Retinal photoreceptors are highly metabolic and their energy consumption is equivalent to that of a multiplying tumor cell. In the present study, we found that PKM2 is the predominant isoform in both rod- and cone-dominant retina, and that it undergoes a light-dependent tyrosine phosphorylation. We also discovered that PKM2 phosphorylation is signaled through photobleaching of rhodopsin. Our findings suggest that phosphoinositide 3-kinase activation promotes PKM2 phosphorylation. Light and tyrosine phosphorylation appear to regulate PKM2 to provide a metabolic advantage to photoreceptor cells, thereby promoting cell survival.

Almost a century ago, Otto Warburg found that the retina had the highest oxygen consumption of any tissue in the body, and he compared retinas to rapidly growing tumor cells^1^,^2^,^3^. The Warburg effect defines a pro-oncogenic metabolism switch such that cancer cells take up more glucose than normal tissue and favor incomplete oxidation of glucose, even in the presence of oxygen^4^. Retinal photoreceptors are highly metabolic and their energy consumption is equivalent to that of a multiplying tumor cell^4^,^5^,^6^,^7^, even though the photoreceptor cells do not divide and are post-mitotic. Nevertheless, both rod and cone photoreceptors shed 10% of their outer segments daily^8^, and these cells must synthesize new lipids, proteins, and nucleic acids to maintain their length and functions. In this regard, the photoreceptor cell is comparable to a rapidly proliferating cancer cell.

In addition, maintenance of the “dark current” requires huge amounts of ATP generated through the Krebs cycle, creating reactive oxygen species (ROS) byproducts. NADPH generated by the pentose phosphate pathway (PPP) is necessary for glutathione recycling for antioxidant protection and for lipid synthesis, as well as for reducing all-*trans*-retinal released by photobleaching of photopigments^9^. Under transient nutrient shortage conditions, photoreceptor mitochondria provide an alternative metabolic pathway for NADPH generation^10^. Since some cells, including photoreceptors, have a great need for NADPH, checkpoints in the glycolytic pathway have been evolutionarily established that allow cells to shunt glucose to the PPP for the generation of NADPH and ribose. This shunting appears to be especially important for photoreceptors due to their large requirement for NADPH for membrane synthesis, antioxidant protection, and all-*trans*-retinal reduction.

Most of the glucose in fetal tissues and cancer cells has been shown to be used for anabolic processes, mainly to synthesize lipids, proteins and nucleic acids. A limited amount of glucose is used for the generation of ATP through oxidative phosphorylation. The switch that redirects glucose to anabolic processes is mediated by a glycolytic enzyme, pyruvate kinase M2 (PKM2). PKM2 is the predominant form of pyruvate kinase expressed in retinal photoreceptors^11^. Inhibition of PKM2 by phosphorylation or decelerating this step leads to the accumulation of phosphoenolpyruvate (PEP), which has been shown to activate the PPP in other tissues^12^,^13^.

PKM2 expression has been documented in the retina^11^; we further characterized the expression of both PKM1 and PKM2 in rod- and cone-dominant retinas. We found that PKM2 undergoes a light-dependent tyrosine phosphorylation in both rods and cones and is signaled in rods through photobleaching of rhodopsin. The tyrosine-105 phosphorylation of PKM2 by oncogenic tyrosine kinases inhibits PKM2 activity by causing the release of its allosteric activator fructose 1,6-bisphosphate (FBP)^14^. We further demonstrated the effects of light on isolated rod and cone photoreceptors. It has been reported recently that PKM2 induces tumor cell growth *via* PI3K/Akt activation^15^, and is up-regulated in PTEN-negative cancer cell lines^16^. Consistent with these earlier findings, we observed that PKM2 phosphorylation is PI3K-dependent.

## Materials and Methods

### Materials

#### Antibodies

Polyclonal pPKM2 (Y105), PKM2, pAkt (S473), and Akt antibodies were obtained from Cell Signaling (Danvers, MA). Rabbit polyclonal anti-red/green cone opsin (M-opsin) antibody was obtained from Millipore (Billerica, MA). α-3 Na/K ATPase antibody was obtained from Novus Biologicals (Littleton, CO). DAPI stain used for nuclear staining and secondary antibodies were purchased from Invitrogen-Molecular Probes (Carlsbad, CA). Monoclonal anti-arrestin antibody was a kind gift from Dr. Paul Hargrave (University of Florida, Gainesville). Monoclonal anti-opsin (1D4) was kindly provided by Dr. Robert Molday (University of British Columbia, Vancouver, Canada).

#### Animals

All animals were treated in accordance with the *ARVO Statement for the Use of Animals in Ophthalmic and Vision Research* and the *NIH Guide for the Care and Use of Laboratory Animals.* The protocols were approved by the IACUC at the University of Oklahoma Health Sciences Center. Animals were born and raised in our vivarium and kept under dim cyclic light (40–60 lux, 12 h light/dark cycle). The *Nrl*^−/−^ mice were kindly provided by Dr. Anand Swaroop (NIH, Bethesda, MD). The Rpe65^−/−^ mice were a kind gift from Dr. Jing-Xing Ma (University of Oklahoma Health Sciences Center, Oklahoma City). We previously reported the generation of *Nrl/cone-p85α* double knockout mice^17^. For light/dark experiments, mice were dark-adapted overnight. The next morning, half of the mice were exposed to normal room light (300 lux equivalent to 3000 R*/rods/sec) for 30 min^18^. Then, the eyes or retinas were harvested after CO_2_ asphyxiation. These tissues were subjected to biochemistry or immunohistochemistry.

#### Chemicals

The OptiPrep™ density gradient was obtained from Sigma (St. Louis, MO). The PI3K inhibitor, LY294002, was procured from Cell Signaling (Danvers, MA). The NADP/NADPH quantification kit (MAK038) was obtained from Sigma. All other reagents were of analytical grade and purchased from Sigma (St. Louis, MO).

### Isolation of photoreceptor cells by OptiPrep™ density gradient centrifugation

We prepared the isolated photoreceptor cells by the method described earlier^19^. Briefly, 14 rod-dominant retinas and 28 cone-dominant *Nrl*^−/−^ retinas, both dark- and light-adapted, were placed in Ringer’s solution [10 mM HEPES (pH 7.4), 130 mM NaCl, 3.6 mM KCl, 12 mM MgCl_2_, 1.2 mM CaCl_2_, and 0.02 mM EDTA] containing 8% OptiPrep™ and gently vortexed for 1 min. We repeated this process five times. The pooled crude lysate was placed on top of the 10–40% OptiPrep™ step gradient. We spun the gradients at 19,210 × g for 60 min at 4 °C. Fractions were collected, and protein localization was examined using immunoblot analysis. We repeated these experiments more than three times. Each time, we observed consistent results, in terms of fractionation and phosphorylation.

### Pyruvate kinase enzyme assay

Lactate dehydrogenase (LDH) coupled enzyme assay was used to measure pyruvate kinase (PK) enzyme activity^20^. The assay was carried out in the presence of 1 μg of either dark- or light-adapted mouse retinal lysate containing an enzyme buffer mixture [50 mM Tris-HCl (pH 7.4), 100 mM KCl, 5 mM MgCl_2_, 1 mM ADP, 0.5 mM PEP, 0.2 mM NADH (reduced form of NAD^+^)] and 8 U of LDH. The PK activity was measured spectrophotometrically by monitoring the reduction in the absorbance at 340 nm from the oxidation of NADH.

### Preparation of tissue for paraffin sectioning using Prefer as a fixative

Prefer solution (Anatech Ltd, Battle Creek, MI) was used to fix the mouse eyes for 15 min at room temperature, followed by 70% ethanol overnight. The tissue was paraffin-embedded, and 5-μm-thick sections were cut and mounted onto slides. These sections were subjected to immunohistochemistry, as described elsewhere^17^. A Nikon Eclipse E800 microscope equipped with a digital camera was used to examine the antibody-labeled complexes. Metamorph (Universal Imaging, West Chester, PA) image analysis software was used to capture images under identical microscope and camera settings.

### Immunoblot analysis

Mouse retinas were homogenized in a lysis buffer containing 1% Triton X-100, 137 mM NaCl, 20 mM Tris-HCl (pH 8.0), 10% glycerol, 1 mM EGTA, 1 mM MgCl_2_, 1 mM phenylmethylsulfonyl fluoride, 0.2 mM Na_3_VO_4_, 10 μg/ml leupeptin, and 1 μg/ml aprotinin^21^. Insoluble material was removed by centrifugation at 17,000 × g for 20 min at 4 °C. The protein concentrations of the solubilized proteins were determined with the bicinchoninic acid reagent, following the manufacturer’s instructions (Pierce Biotechnology, Rockford, IL). Ten micrograms of retinal proteins or 1–10 μl of OptiPrep™ density gradient fractions were run on 10% SDS-PAGE, followed by protein blotting onto nitrocellulose membranes. After blocking the membranes with 5% non-fat dry milk power (Bio-Rad) or 5% bovine serum albumin (Sigma) for 45–60 min at room temperature, blots were incubated with anti-opsin (1:10,000), anti-pPKM2 (1:1000), anti-PKM2 (1:1000), anti-pAkt (1:1000), anti-Akt (1:1000), and anti-α3-Na/K ATPase (1:1000) overnight at 4 °C. Then, the blots were washed and incubated with HRP-coupled anti-mouse or anti-rabbit secondary antibodies for 60 min at room temperature. After washing, blots were developed with enhanced SuperSignal™ West Dura Extended Duration Substrate (Thermo Fisher Scientific, Waltham, MA) and visualized using a Kodak Imager with chemiluminescence capability.

## Results

### Localization of PKM2 and PKM1 in rod- and cone-dominant retinas

**Figure 1:**
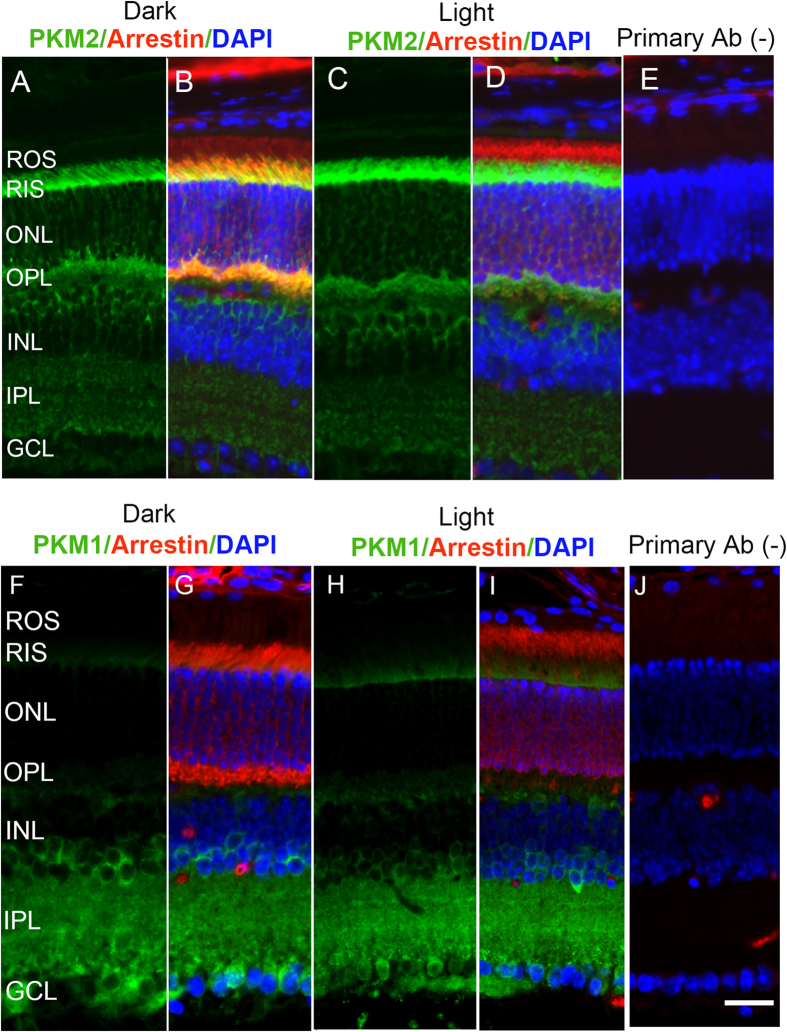
Immunofluorescence analysis of PKM2 and PKM1 in mouse retinas. Prefer-fixed sections of dark- (**A,B,F,G**) and light-adapted (**C,D,H,I**) mouse retinas were stained for PKM2 (**A–D**), PKM1 (**F–I**), arrestin (**B,D,G,I**) and DAPI (**B,D,E,G,I,J**). Panels (**B,D,G**,**I)** represent the merged images of either PKM2 or PKM1 and arrestin, whereas panels (**E**,**J)** represent the omission of primary antibody. ROS, rod outer segments; RIS, rod inner segments; ONL, outer nuclear layer; OPL, outer plexiform layer; INL, inner nuclear layer; IPL, inner plexiform layer; GCL, ganglion cell layer. Scale bar 50 μm.

Retinal sections from dark- and light-adapted Balb/c (rod-dominant) and *Nrl*^−/−^ (cone-dominant) mice were stained with PKM1 and PKM2 antibodies. Arrestin immunolocalization was used to confirm the adaptability of mice to dark- and light-adapted conditions. Under dark-adapted conditions, arrestin predominantly localized to rod inner segments and the outer plexiform layer (Fig. 1B,G). Upon light illumination, arrestin translocated to rod outer segments (Fig. 1D,I). Our results suggest that PKM2 expression was predominantly localized to rod inner segments and the outer plexiform layer in both dark- and light-adapted conditions (Fig. 1A,C). We did not observe any change in the localization of PKM2 under dark- or light-adapted conditions. We found that PKM1 was predominantly expressed in the rod inner plexiform layer and ganglion cell layer; some expression was also found in rod inner segments under both dark- and light-adapted conditions (Fig. 1F,H). The localization of PKM1 remained the same, irrespective of dark- or light-adapted conditions. These experiments suggest that both PKM2 and PKM1 are expressed in rod photoreceptor cells, and that PKM2 could be the predominant isoform in rods.

**Figure 2:**
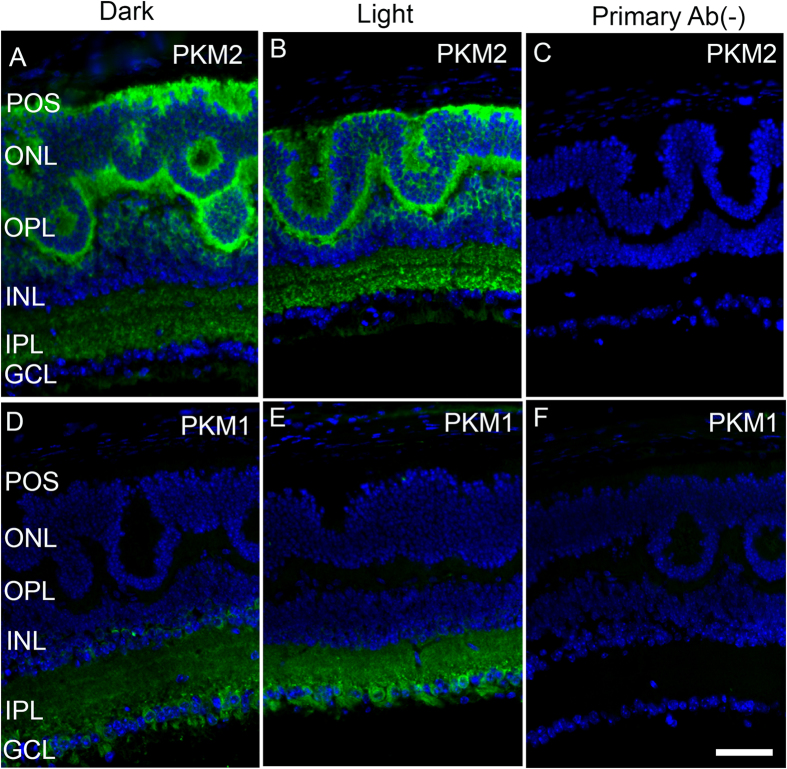
Immunofluorescence analysis of PKM2 and PKM1 in cone-dominant retinas. Prefer-fixed sections of dark- (**A,D**) and light-adapted (**B,E**) *Nrl*^−/−^ mouse retinas were subjected to immunofluorescence with anti-PKM2 (**A,B**) and anti-PKM1 (**D,E**). Panels (**C,F**) represent the omission of primary antibody. POS, photoreceptor outer segments; ONL, outer nuclear layer; OPL, outer plexiform layer; INL, inner nuclear layer; IPL, inner plexiform layer; GCL, ganglion cell layer. Scale bar 50 μm.

To determine the expression of PKM2 in cones, we took the advantage of *Nrl*^−/−^ mice as we cannot study their expression in the wild type mouse retina since it contains only 3–5% of cones. The *Nrl*^−/−^ mice do not express rods due to a block in the differentiation of rod precursor cells, which results in the expression of all cone-like cells that are indistinguishable biochemically from normal cones^22^,^23^,^24^,^25^. The *Nrl*^−/−^ mouse retina has a characteristic feature of large undulations of the outer nuclear layer (ONL), commonly known as rosettes. These rosettes arise due to defects in the outer limiting membrane and delayed maturation of a subset of photoreceptors^26^. To determine the expression of pyruvate kinase isoforms, *Nrl*^−/−^ mouse retinal sections stained with PKM2 and PKM1 antibodies showed that PKM2 was predominantly expressed in photoreceptor inner segments and the outer plexiform layer; a weak immunoreactivity was also observed in the inner plexiform layer under both dark- and light-adapted conditions (Fig. 2A,B). PKM1 expression was predominantly localized to the inner plexiform and ganglion cell layers under both dark- and light-adapted conditions (Fig. 2D,E). Our immunohistochemistry analyses showed no PKM1 expression in the photoreceptor layer (Fig. 2D,E). These observations suggest that PKM2 could be the major isoform in cone photoreceptor cells.

### PKM2 undergoes a light-dependent tyrosine phosphorylation in the retina

**Figure 3:**
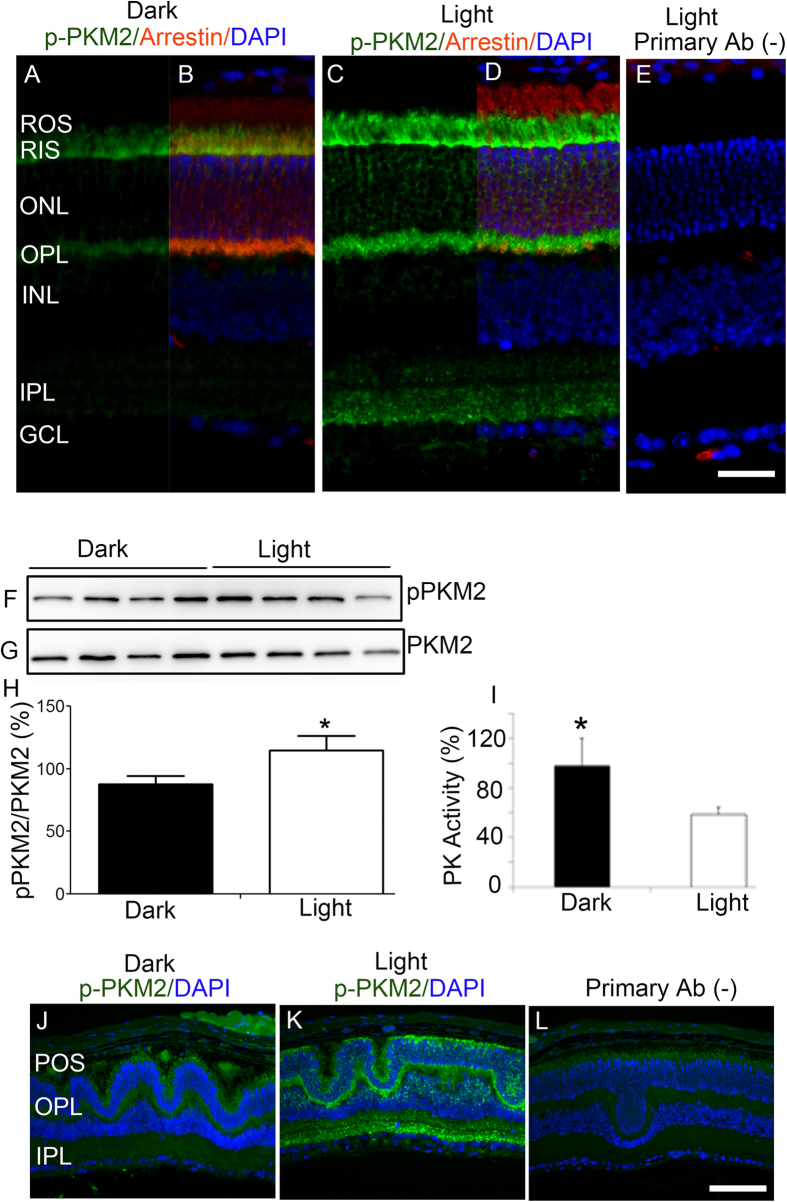
Light-dependent PKM2 phosphorylation in rod- and cone-dominant retinas. Prefer-fixed sections of dark- (**A,B**) and light-adapted (**C,D**) mouse retinas were subjected to immunofluorescence with anti-p-PKM2 (Y105) (**A,C**), and anti-arrestin (**B,D**) antibodies. Panels (**B,D)** represent the merged images of p-PKM2 and arrestin, whereas panel (**E)** represents the omission of primary antibody. ROS, rod outer segments; RIS, rod inner segments; ONL, outer nuclear layer; OPL, outer plexiform layer; INL, inner nuclear layer; IPL, inner plexiform layer; GCL, ganglion cell layer. Retinal lysates from dark- and light-adapted mice were subjected to immunoblot analysis with anti-pPKM2 (**F**) and anti-PKM2 (**G**) antibodies. Densitometric analysis of pPKM2 was performed in the linear range of detection, and absolute values were then normalized to PKM2 (**H**). Data are mean + *SEM, n* = 4. *p < 0.05. Pyruvate kinase activity was measured from dark- and light-adapted mouse retinas with an LDH-coupled enzyme assay. Data are mean ± *SD, n* = 3, *p < 0.05. Prefer-fixed sections of dark- (**J**) and light-adapted (**K**) *Nrl*^−/−^ mouse retinas were subjected to immunofluorescence with anti-pPKM2 (**J,K**). Panel (**L**) represents the omission of primary antibody. POS, photoreceptor outer segments; OPL, outer plexiform; IPL, inner plexiform layer. Scale bar 50 μm. Full-length blots are presented in the Supplementary Information.

In tumor cells, PKM2 undergoes tyrosine phosphorylation (Y105), which results in the inhibition of PKM2 activity. This regulation of signaling diverts glucose metabolites from energy production to anabolic processes (protein, lipid, and nucleic acid synthesis), in addition to generating NADPH to support antioxidant metabolism^27^. To determine whether PKM2 undergoes tyrosine phosphorylation in the retina, retinal sections from dark- and light-adapted mice were subjected to immunohistochemistry with phospho-specific PKM2-Y105 and arrestin antibodies. The results revealed phospho-PMK2 immunoreactivity in dark-adapted retina (Fig. 3A,B): phosphorylation was enhanced under light-adapted conditions, especially in rod inner segments and the outer and inner plexiform layers (Fig. 3C,D). To quantify the PKM2-Y105 phosphorylation, retinal lysate was subjected to immunoblot analysis with anti-PKM2-Y105 and anti-PKM2 antibodies. These results suggest a significant phosphorylation of PKM2 in light-adapted retinas compared with dark-adapted retinas (Fig. 3F–H).

To determine whether tyrosine phosphorylation of PKM2 has an effect on catalytic activity, we measured pyruvate kinase activity in light and dark-adapted retinas and found a significant reduction in pyruvate kinase activity in light-adapted retinas (Fig. 3I). *Nrl*^−/−^ mouse retinal sections stained with PKM2-Y105 antibody also showed light-dependent tyrosine phosphorylation in photoreceptor inner segments and the outer plexiform and inner plexiform layers in cone-dominant retinas (Fig. 3J–L). These observations suggest that PKM2 undergoes a light-dependent tyrosine phosphorylation in cone-dominant retina.

### Rhodopsin photobleaching regulates the phosphorylation state of PKM2

**Figure 4:**
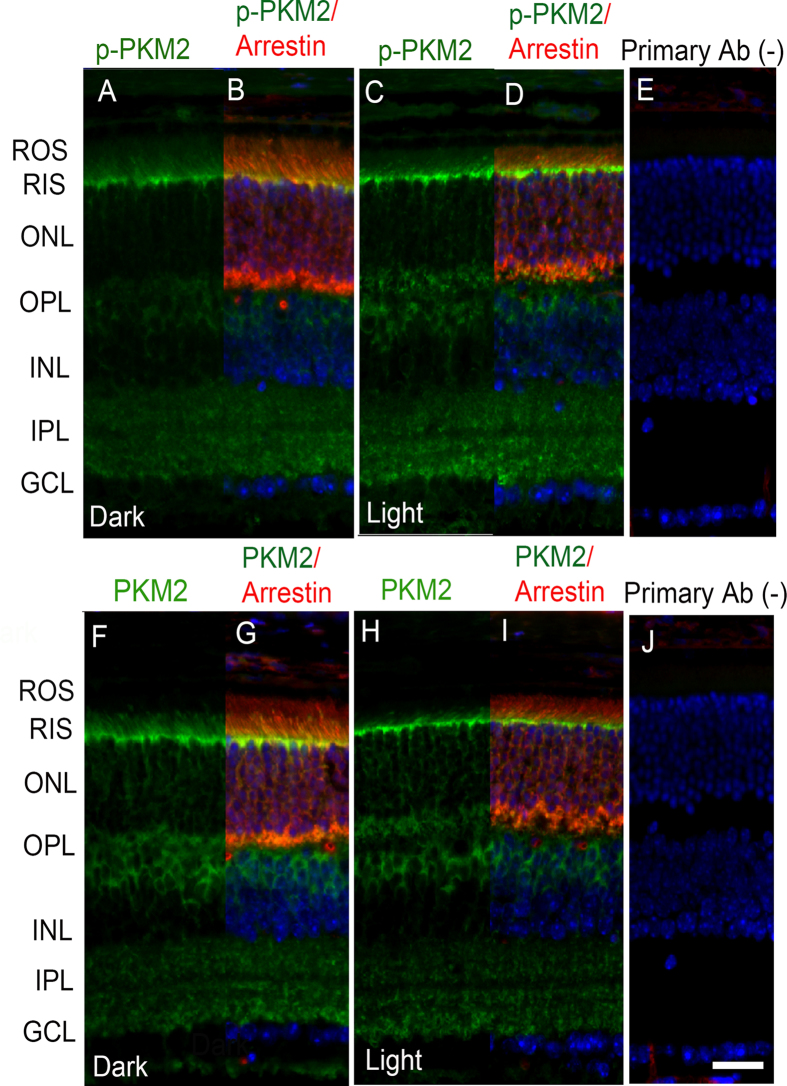
Rhodopsin activation regulates the phosphorylation of PKM2. Prefer-fixed sections of dark- (**A,B,F,G**) and light-adapted (**C,D,H,I**) *Rpe65*^−/−^ mouse retinas were subjected to immunofluorescence with anti-pPKM2 (**A–D**), anti-PKM2 (**F–I**), and arrestin (**B**,**D,G,I**) antibodies. Panels (**E,J**) represent the omission of primary antibody. ROS, rod outer segments; RIS, rod inner segments; ONL, outer nuclear layer; OPL, outer plexiform layer; INL, inner nuclear layer; IPL, inner plexiform layer; GCL, ganglion cell layer. Scale bar 50 μm.

To determine whether the observed light-dependent tyrosine phosphorylation of PKM2 signals through rhodopsin activation, we used the *Rpe65*^−/−^ mouse model. Loss of retinal pigment epithelium 65 protein (Rpe65) in the retinal pigment epithelium (RPE) results in the absence of 11*-cis*-retinal regeneration, leading to the absence of rhodopsin photobleaching or activation^28^. It has been shown that, in *Rpe65*^−/−^ mice, arrestin does not translocate to outer segments under light-illumination^29^, and we found similar results (Fig. 4). In these retinas, we did not observe a light-dependent enhancement of PKM2 phosphorylation (Fig. 4A,C). We found that PKM2 expression was less (Fig. 4F,H) than that in wild-type retina (Fig. 1A,C). These observations suggest that rhodopsin signaling may regulate the phosphorylation state of PKM2.

### Biochemical characterization of PKM2 and PKM1 isoforms in isolated rod and cone photoreceptor cells

**Figure 5:**
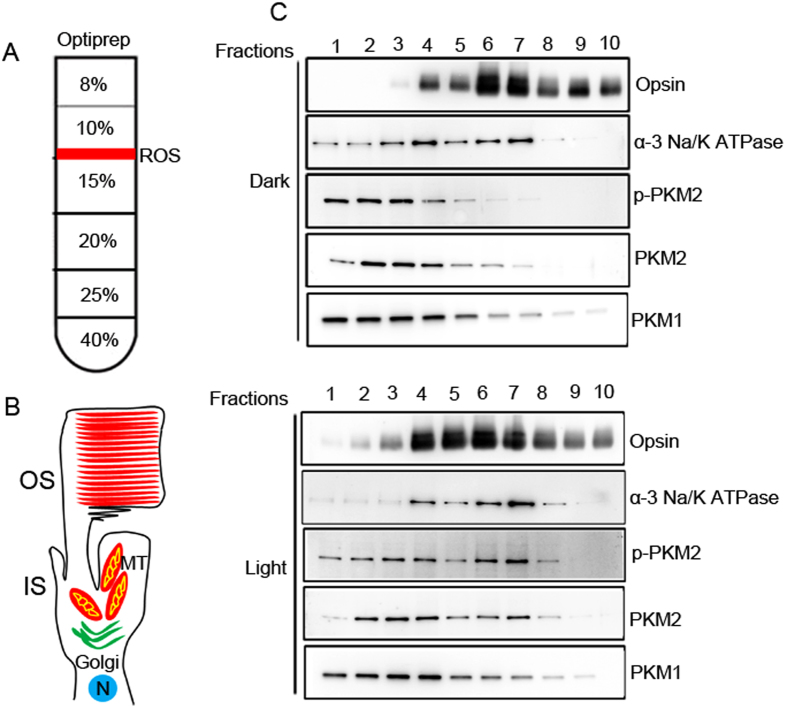
Biochemical characterization of PKM2 and PKM1 isoforms on isolated photoreceptor cells from dark- and light-adapted Balb/c mice. Retinal homogenates from dark- and light-adapted Balb/c mice were subjected to OptiPrep™ (8–40%) density gradient centrifugation (**A**). Fractions of inner segments and intact photoreceptors (**B**) were collected from the top to the bottom of the gradients. A ten-microliter sample (one-microliter for rhodopsin) was subjected to immunoblot analysis (**C**) with opsin, α-3 Na/K ATPase, pPKM2, PKM2, and PKM1 antibodies. Full-length blots are presented in the Supplementary Information.

Biochemical analyses were performed on isolated cellular fragments from mouse retinas on an OptiPrep^TM^ density gradient (8–40%) that yields rod outer segments with large portions of the rod inner segments attached (Fig. 5A,B). Retinas from Balb/c mice and *Nrl*^−/−^ mice were subjected to density gradient centrifugation, and fractions were collected. We used the most predominant rod outer segment resident marker, rhodopsin, for rods. For cones, M-opsin was used. It has been shown that cells break open and release some portion of the cytoplasm, including soluble proteins, during sample preparation. The breakpoint occurs in the inner segment, while the soluble components are essentially retained within the outer segments^19^.

We also used an inner segment marker of α-3 Na/K ATPase to identify intact photoreceptors that are positive for both rhodopsin and α-3 Na/K ATPase, and inner segments that are positive only for α-3 Na/K ATPase. Fractionation by density gradient ultracentrifugation revealed evidence of both intact rod outer and inner segments, based on the immunoreactivity of rhodopsin and α-3 Na/K ATPase (Fig. 5C). Our data also suggest that there was no difference in the fractions pattern of rhodopsin-positive cells in dark- or light-adapted conditions (Fig. 5C). However, we found two peaks of α-3 Na/K ATPase; one peak co-migrated with rhodopsin (intact rod photoreceptor). The other earlier peak was suggestive of rod inner segments and was indistinguishable in dark- or light-adapted conditions. PKM1 was predominantly localized to the rod inner segments, and some fraction of PKM1 co-migrated with rhodopsin, irrespective of dark- or light-adaptation (Fig. 5C). Under dark-adapted conditions, PKM2 was predominantly localized to rod inner segments; however, PKM2 co-migrated with peak rhodopsin fractions under light-adapted conditions (Fig. 5C). This experiment does not rule out the possibility that PKM2 has a higher affinity for light-adapted photoreceptor membranes.

**Figure 6:**
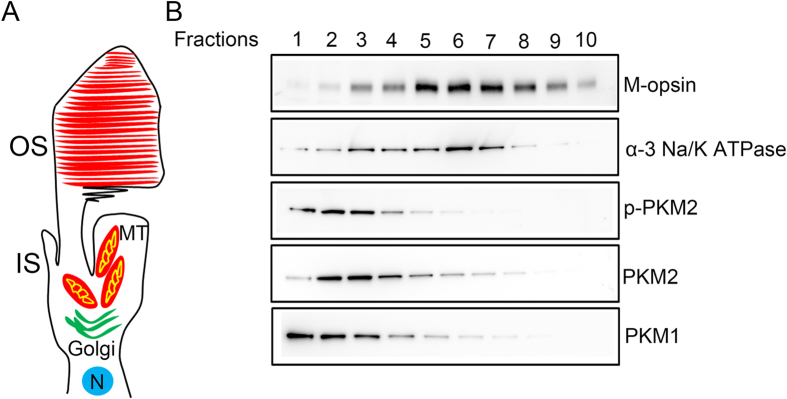
Biochemical characterization of PKM2 and PKM1 isoforms on isolated cone photoreceptor cells from *Nrl*^−/−^ mice. Retinal homogenates from *Nrl*^−/−^ mice were subjected to OptiPrep™ (8–40%) density gradient centrifugation, and fractions of inner segments and intact photoreceptors (**A**) were collected from the top to the bottom of the gradients. A ten-microliter sample (three-microliter for M-opsin) was subjected to immunoblot analysis (**B**) with M-opsin, α-3 Na/K ATPase, pPKM2, PKM2, and PKM1 antibodies. Full-length blots are presented in the Supplementary Information.

Fractionation of cone-dominant *Nrl*^−/−^ retinas after OptiPrep^TM^ density gradient centrifugation revealed that an M-opsin peak co-migrated with α-3 Na/K ATPase, suggesting intact cone photoreceptor cells (Fig. 6). Immunoblot analysis of PKM1 and PKM2 indicated the presence of both isoforms in cones (Fig. 6). We also observed PKM2 phosphorylation in cone photoreceptors (Fig. 6). Taken together, the results of these experiments suggest that the PKM2 and PKM1 isoforms are expressed in both rods and cones.

### Biochemical characterization of PKM2 and PKM1 isoforms in isolated photoreceptors from *Rpe65* ^−/−^ mice

**Figure 7:**
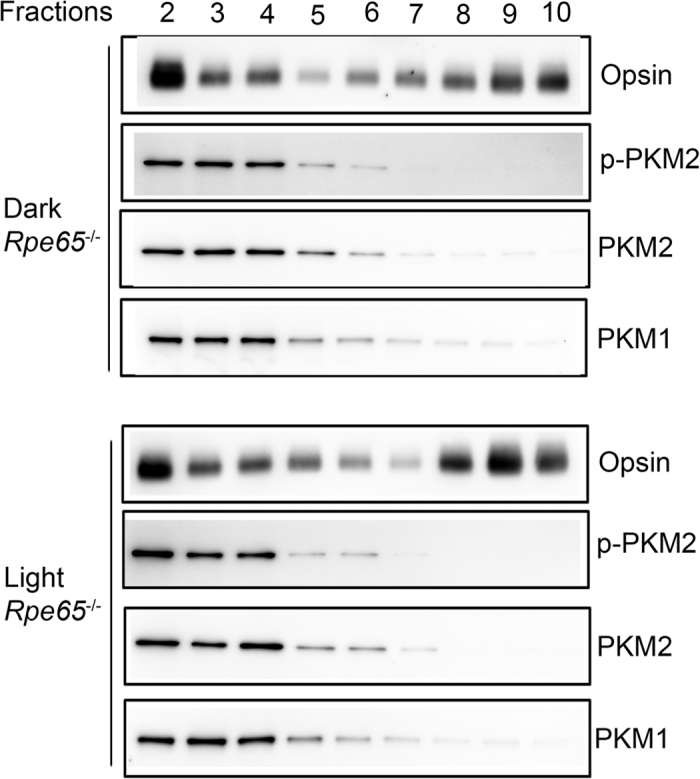
Biochemical characterization of PKM2 and PKM1 isoforms on isolated photoreceptor cells from dark- and light-adapted *Rpe65*^−/−^ mice. Retinal homogenates from dark- and light-adapted *Rpe65*^−/−^ mice were subjected to OptiPrep (8–40%) density gradient centrifugation, and fractions were collected from top to the bottom of the gradients. A ten-microliter sample (one-microliter for opsin) was subjected to immunoblot analysis with opsin, pPKM2, PKM2, and PKM1 antibodies. Full-length blots are presented in the Supplementary Information.

Fractionation of dark- and light-adapted retinas from *Rpe65*^−/−^ mice after OptiPrep^TM^ density gradient centrifugation showed a distinct pattern of opsin migration compared with that of wild-type retinas (Fig. 7). The results revealed no opsin peak, and that opsin was distributed throughout the fractions, irrespective of dark- or light-adaptation (Fig. 7). There was also a significant portion of opsin in the inner segment area (Fig. 7). PKM1 and PKM2 localization remained the same in both dark- and light-adapted conditions (Fig. 7). In addition, the phosphorylation state of PKM2 was unchanged under dark- or light-adapted conditions (Fig. 7). These experiments further support the findings from immunohistochemical studies indicating that PKM2 phosphorylation is regulated through rhodopsin photoactivation.

### PKM2 phosphorylation is under the control of phosphoinositide 3-kinase (PI3K)

**Figure 8:**
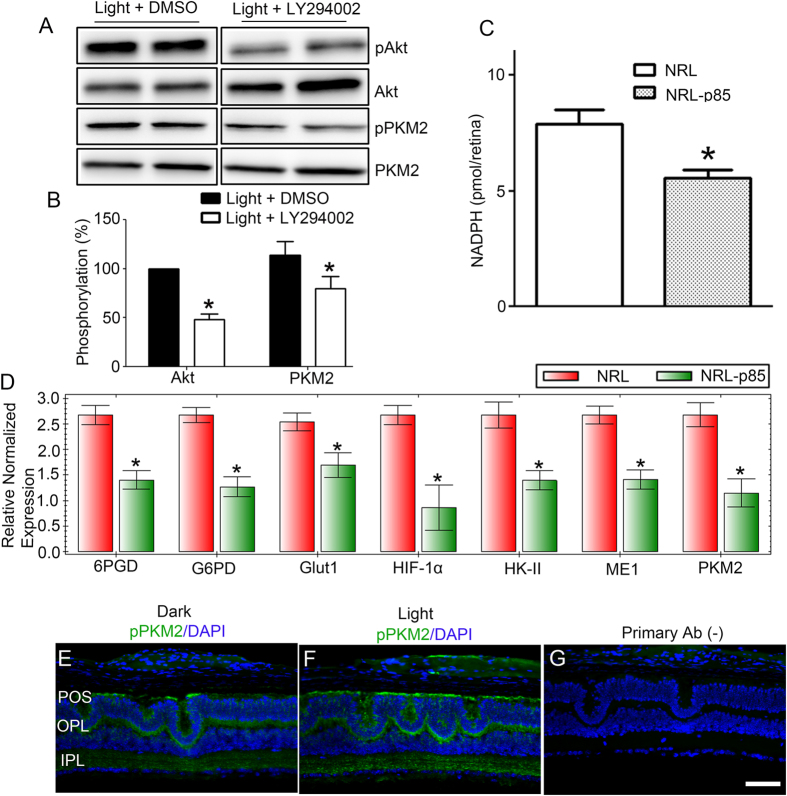
Light-induced PKM2 phosphorylation is PI3K-dependent. *Ex vivo* mouse retinal explants prepared in the dark were incubated in DMSO or PI3K-inhibitor LY294002 for 10 min prior to exposure to room light for 30 min. Retinal proteins were immunoblotted with anti-pAkt, anti-Akt, anti-p-PKM2, and anti-PKM2 (**A**) antibodies. Densitometric analysis of pAkt/Akt and pPKM2/PKM2 (**B**). Data are mean + *SEM, n* = 4. *p < 0.05. *Nrl*^−/−^ (NRL) and *Nrl*^−/−^*/cone-p85α*^−/−^ (NRL-p85) mouse retinas were used to measure NADPH levels (**C**). Data are mean + *SEM, n* = 4. *p < 0.02. Equal amounts of retinal mRNA from three independent one-month-old Nrl^−/−^ and *Nrl*^−/−^*/cones-p85α*^−/−^ mice were used for real-time (RT)-PCR and normalized by β-actin levels (**D**). The mRNA levels were averaged for 6PGD, G6PD, Glut1, HIF-1α, HK-II, ME1, and PKM2. The data are mean ± *SD, n* = 3. *P < 0.05. Decreased phosphorylation of PKM2 in cones lacking the p85α subunit of PI3K. Prefer-fixed sections of dark- (**E**) and light-adapted (**F**) *Nrl*^−/−^*/cone-p85α*^−/−^ mouse retinas were subjected to immunofluorescence with anti-pPKM2. Panel **G** represents the omission of primary antibody. POS, photoreceptor outer segments; OPL, outer plexiform layer; IPL, inner plexiform layer. Scale bar 50 μm. Full-length blots are presented in the Supplementary Information.

We previously reported light-dependent PI3K activation in both rods and cones^18^,^30^. To identify early pathways that are both stimulated by light and are PI3K-dependent, we treated retinal *ex vivo* implants with PI3K inhibitor (LY294002) or DMSO (vehicle), followed by light exposure. Proteins that differentially migrated on 2D-electophoresis were identified using mass spectrometer analysis. We found that the differentially migrated proteins belonged to glycolytic and energy metabolism pathways, which also include pyruvate kinase (data not shown). To determine whether PKM2 phosphorylation is under the control of PI3K activation, we incubated *ex vivo* mouse retinal explants prepared in the dark in DMSO or PI3K-inhibitor (LY294002) for 10 min before exposing them to room light for 30 min. Light-dependent phosphorylation of both Akt (downstream effector of PI3K) and PKM2 was reduced in the presence of PI3K inhibitor (Fig. 8A,B). These observations show that PKM2 phosphorylation is both light- and PI3K-dependent and establish an important link between photon capture in the outer segments and glucose metabolism in the inner segments.

### Reduced NADPH levels in cone p85α^−/−^/Nrl^−/−^ double knockout mouse retina

It has been reported recently that PKM2 induces tumor cell growth *via* PI3K/Akt activation^15^. In cancer cells, decreased PKM2 activity due to phosphorylation results in increased NADPH production^12^. To determine whether loss of PI3K decreases the production of NADPH, we measured NADPH levels in cone-dominant *Nrl*^−/−^ and Nrl^−/−^*/p85* subunit of PI3K KO mouse retinas. We observed significantly lower levels of NADPH generation in *Nrl*^−/−^*/p85* KO retinas than in *Nrl*^−/−^ retinas (Fig. 8C). This finding suggests that PI3K may regulate PKM2 activity and subsequent generation of NADPH.

### Decreased expression of genes involved in glycolysis and the pentose phosphate pathway (PPP) in *Nrl* ^−/−^ */cone-p85α* ^−/−^ retinas

To determine whether loss of PI3K in cones affects the expression of key metabolic target genes that increase NADPH production, we conducted real-time PCR analysis using the primers^31^ described in Table 1. In *Nrl*^−/−^ and *Nrl*^−/−^*/cone-p85α*^−/−^ retinas, we analyzed the expression of the gene that increases glucose uptake, glucose transporter 1 (Glut1); the kinase that phosphorylates glucose, hexokinase II (HK-II); and the two key enzymes that shunt glucose into PPP for NADPH generation, glucose 6-phosphate dehydrogenase (G6PD)^32^ and 6-phosphogluconate dehydrogenase (GPGD). Our results suggest that all genes involved in glycolysis and the PPP shunt were significantly decreased relative to β-actin in *Nrl*^−/−^*/cone-p85α*^−/−^ retinas compared with *Nrl*^−/−^ retinas (Fig. 8D). We also found reduced expression of other glycolytic genes, PKM2 and malic enzyme 1 (ME1), in *Nrl*^−/−^*/cone-p85α*^−/−^ retinas (Fig. 8D), which also contribute to an upsurge in NADPH production^14^. In *Nrl*^−/−^*/cone-p85α*^−/−^ retinas, we found reduced expression of hypoxia-inducible factor 1 α (HIF-1α), which regulates the transcription of many glycolytic genes (Fig. 8D). Our data suggest that glucose uptake, preservation, and divergence into the PPP are reduced in PI3K-deficient cones. Consistent with these observations, the phosphorylation of PKM2 is significantly reduced in *Nrl*^−/−^*/cone-p85α*^−/−^ retinas (Fig. 8F) compared with light-exposed *Nrl*^−/−^ retinas (Fig. 3K). These observations suggest that PI3K may regulate the phosphorylation state of PKM2.

## Discussion

Studies from our laboratory and others have shown that photoreceptors have active oncogenic signaling pathways^11^,^17^,^33^,^34^,^35^. Very recently, we reported that activation of oncogenic tyrosine kinase signaling promotes cone photoreceptor survival mediated through insulin receptor activation^30^. In terms of energy utilization and activation of tyrosine kinase signaling pathways, photoreceptors behave like cancer cells.

The glycolytic enzyme, PKM2 has been previously shown to be a phosphotyrosine-binding protein^27^. In tumor cells, PKM2 undergoes tyrosine phosphorylation on tyrosine 105 (Y105)^36^. It has also been shown that tyrosine phosphorylation inhibits pyruvate kinase (PK) activity in cancer cells^36^. Further, PK triggers a metabolic feedback loop that controls redox metabolism in respiring cells^13^. In yeast, low PK levels resulted in the accumulation of PK substrate PEP^13^. PEP acts as a feedback inhibitor of triosephosphate isomerase (TPI)^13^. TPI inhibition activates the PPP, which increases antioxidant metabolism and prevents reactive oxygen species accumulation^13^. The NADPH generated through this system is pivotal for lipid synthesis and antioxidant metabolism^13^. However, their study did not show that PKM2 is the rate-limiting enzyme, nor do they indicate how much phosphorylation inhibits PK activity in yeast cells^13^. The idea that inhibition of PKM2 can enhance PPP activity is only a hypothesis that is yet to be tested in cancer or photoreceptor cells. For the hypothesis that PK activity is the rate-limiting (slowest) step in glycolysis to be correct, one would have to show that phosphorylation of PKM2 brings PK activity below the activity of enolase to make it rate-limiting. To date, neither experiment has been conducted.

In photoreceptor cells, NADPH is required for *de novo* synthesis of fatty acids (components of newly synthesized membrane phospholipids), reduction of GSSG to GSH (the substrate for many of the cellular thioreductases necessary for maintaining a reducing environment in the cell), and detoxification of all-*trans*-retinal (produced in the light) by retinol dehydrogenase^9^. Similar to cancer cells, we also observed the tyrosine phosphorylation of PKM2 in rod and cone photoreceptor cells. Interestingly, light drives further increases the phosphorylation of PKM2. This may be due to the activation of tyrosine kinase signaling proteins that promote PKM2 phosphorylation. Consistent with this idea, our laboratory previously reported a light-dependent activation of several oncogenic signaling proteins, including insulin receptor, non-receptor tyrosine kinases, phosphoinositide 3-kinase, and Akt in photoreceptor cells^18^,^30^,^37^. We have observed no effect of light on the activation of oncogenic receptor and non-receptor tyrosine kinases in mouse retinas that are deficient in rhodopsin photobleaching^30^,^38^,^39^, further establishing the role of photobleachable visual pigments in this process.

The functional consequence of enhanced light-dependent tyrosine phosphorylation of PKM2 is unknown. In the present study, we showed that light increases the level of phosphorylation by about 40%, and causes an approximate 50% reduction in enzyme activity. However, there is no interpretable quantitative relationship between these numbers. Due to the limitation of reagents, we cannot quantify the percentage of the protein that is phosphorylated. The ideal way to quantify the PKM2 phosphorylation under dark- and light-adapted conditions would be expression and purification of PKM2, followed by *in vitro* phosphorylation by FGF1-R. Running a standard curve with different amounts of phospho-PKM2, we could measure the phospho-signal by ELISA using phospho-PKM2 antibody. These studies are underway in our laboratory.

In the present study, we found reduced phosphorylation of PKM2 in *Rpe65*^−/−^ mouse retinas, a mouse model that lacks rhodopsin photobleaching due to an inability to regenerate the chromophore, 11*-cis*-retinal^28^. These observations suggest a regulatory role of the tyrosine kinase signaling pathway in facilitating PKM2 phosphorylation through rhodopsin activation. In tumor cells, oncogenic forms of fibroblast growth factor receptor type 1 (FGFR1) have been shown to inhibit the PKM2 isoform’s activity through a direct phosphorylation of PKM2 tyrosine residue 105^36^. In the present study, we did not examine the effect of FGFR1 on PKM2 phosphorylation in photoreceptor cells; however, we found a light-dependent decrease in the activity of PK in light-adapted versus dark-adapted conditions. Light- and growth factor receptor-mediated effects on energy metabolism are not uncommon in the retina, as we previously reported a light- and IR/PI3K/Akt-dependent association of hexokinase II (HK-II) and a mitochondrial transmembrane protein involved with ATP secretion, the voltage-dependent anion channel (VDAC)^40^. This association enhances HK-II phosphorylation of glucose (Glu) to glucose-6-P (Glu-6-P)^32^, the initial step in glycolysis.

Our biochemical characterization of pyruvate kinase in isolated rod and cone photoreceptors clearly shows the expression of both PKM1 and PKM2 isoforms. It is interesting to note that PKM1 isoform localization remains the same under both dark- and light-adapted conditions, whereas, there are distinct differences in PKM2 localization in rods isolated under dark- and light-adapted conditions. Under light-adapted conditions, we found increased PKM2 phosphorylation in fractions that co-eluted with rhodopsin, a phenomenon that is lost in mice lacking bleachable rhodopsin. Studies to examine whether PKM2 has increased affinity for light-adapted membranes upon phosphorylation, or whether phosphorylation causes PKM2 translocation between rod inner and outer segments, are needed.

Interestingly, we observed that PKM2 phosphorylation is PI3K-dependent. Loss of PI3K in cones resulted in decreased NADPH production and reduced expression of genes involved in glucose uptake, preservation, and divergence. In this experiment, phosphorylation of PKM2 may or may not be what is causing the lower levels of NADPH. PI3K is known to regulate glucose uptake and Glut1 glucose transporter expression. Further studies are required to examine the role of phosphorylation of PKM2 by generating phospho-mutant of PKM2 (Y105F) and examining NADPH production in the presence of a functional PI3K. We have not performed experiments on rods lacking the p85α subunit of PI3K. When we deleted p85α in rods, we did not observe any retinal phenotype due to the expression of p85β in rods^41^. However, p85α deletion in cones resulted in age-related cone degeneration^35^. To separate the specific PI3K signaling and its effect on metabolic genes in cones from rods, we generated cone-*p85α/N*rl double knockout mice^17^, and carried out the experiments described in Fig. 8. PI3K exists in three classes: Class I, Class II, and Class III. The p85α subunit belongs to class I PI3K, and its deletion has no effect on rods. Deletion of class III PI3K in rods has been shown to result in age-related rod degeneration^42^. Studies to examine the effect of class III PI3K on PKM2 phosphorylation are currently underway in our laboratory. It has also been reported that PKM2 induces tumor cell growth *via* PI3K/Akt activation^15^ and is up-regulated in PTEN-negative cancer cell lines^16^. PTEN is a phosphatase that inactivates PI3K signaling^43^. In the retina, deletion of PTEN prolongs cone survival in animal models of retinitis pigmentosa (RP)^31^. We previously reported that ablation of class I PI3K in cones resulted in age-related cone degeneration^35^,^44^. Based on our current data, PKM2 may be an attractive target for therapeutic intervention.

In addition to phosphorylation, PKM2 has been shown to undergo acetylation on K305^45^, and oxidation of cysteine-358^46^. Both of these modifications inhibit PKM2 activity, which results in diverting glucose flux into anabolic PPP, thereby generating sufficient reducing potential for detoxification of reactive oxygen species^46^. Activation of PKM2 phosphorylation might be useful in protecting the dying retinal cells. It has recently been shown that PKM2 is a novel protein tyrosine phosphate 1B (PTP1B) substrate, and PTP1B deficiency has been shown to lead to increased Y105-PKM2 phosphorylation both *in vitro* and *in vivo*^47^. Further, pharmacological inhibition of PTP1B increased Y105-PKM2 phosphorylation and decreased PKM2 activity^47^. We previously reported that either pharmacological inhibition of PTP1B^48^ or conditional ablation of PTP1B in a mouse model of cone degeneration is neuroprotective^30^. Studies to establish the link between the activation of PKM2 phosphorylation and protection of photoreceptor degeneration are ongoing in our laboratories.

Recent works suggest non-canonical (non-metabolic) function of PKM2 serves to regulate gene expression, and these effects are based on PKM2 protein-kinase function or protein-protein interactions involving PKM2^49^. PKM2 has been shown to be a protein kinase that catalyzes the transfer of phosphate group directly from PEP to serine, threonine, or tyrosine residues on various protein substrates^49^. It has been shown that PKM2 is competitively inhibited by ADP, which suggests the accommodation of binding of multiple phosphate acceptors in its active site, including ADP, target proteins, and the phosphate donor PEP^49^. It is not clear how PKM2 selectively chooses nucleotide-diphosphate substrates while accepting protein substrates in the same site^49^. More than 200 proteins have been identified as targets of PKM2^50^. Using purified constituents, the ability of PKM2 to phosphorylate protein substrates accepting either PEP or ATP as a phosphate donor has been challenged^51^.

PKM2, but not PKM1, has been reported to activate gene expression by binding and transactivating the transcriptional factor HIF1α^52^, which is known to regulate the transcription of many glycolytic genes. It is more likely that alterations in these enzymes, potentially a direct consequence of reduced HIF1α expression, alters NADPH levels in p85 deficient retinas. It may be possible that phosphorylated state of PKM2 has a regulatory effect on HIF1α. Consistent with idea, we observed increased phospho-PKM2 immunoreactivity in the outer nuclear layer of the light-adapted mouse retina compared to the dark-adapted retina (Fig. 3A–D). Further studies are required to establish this link.

## Additional Information

**How to cite this article**: Rajala, R. V. S. *et al*. The Warburg Effect Mediator Pyruvate Kinase M2 Expression and Regulation in the Retina. *Sci. Rep.* **6**, 37727; doi: 10.1038/srep37727 (2016).

**Publisher’s note:** Springer Nature remains neutral with regard to jurisdictional claims in published maps and institutional affiliations.

## Supplementary Material

Supplementary Information

## Figures and Tables

**Table 1:** Real-time PCR primers used to measure gene expression.

Gene	Forward Primer	Reverse Primer
Hif1α	GATGACGGCGACATGGTTTAC	CTCACTGGGCCATTTCTGTGT
Glut1	TCAACACGGCCTTCACTG	CACGATGCTCAGATAGGACATC
HK-II	GGAACCCAGCTGTTTGACCA	CAGGGGAACGAGAAGGTGAAA
6PGD	AGACAGGCAGCCACTGAGTT	AAGTTCTGGGTTTCGCTCAA
G6PD	CCTACCATCTGGTGGCTGTT	TGGCTTTAAAGAAGGGCTCA
PKM2	ATTGCCCGAGAGGCAGAGGC	ATCAAGGTACAGGCACTACACGCAT
ME1	AGAGGTGTTTGCCCATGAAC	GCTGGTCGGATTACTCAAAGC
β-actin	ACTGGGACGACATGGAGAAG	GGGGTGTTGAAGGTCTCAAA
