# Supplementary material for: The Warburg Effect Mediator Pyruvate Kinase M2 Expression and Regulation in the Retina
Source: Sci Rep. 2016 Nov 24;6:37727. doi: 10.1038/srep37727 (PMC5121888; doi:10.1038/srep37727)
Supplement: Supplementary Information [file srep37727-s1.pdf]

The Warburg Effect Mediator Pyruvate Kinase M2 Expression and Regulation in the Retina

Raju V.S. Rajala[1, 2,3,4,5], Ammaji Rajala[1, 4], Christopher Kooker[1, 4, 5], Yuhong Wang[1, 4], and Robert E. Anderson[1, 3, 4, 5]

From the Departments of [1]Ophthalmology, [2]Physiology, and [3]Cell Biology, University of Oklahoma Health Sciences Center, Oklahoma City, OK; [4]Dean McGee Eye Institute, [5]Oklahoma Center for Neuroscience, Oklahoma City, OK, USA

Figure 3

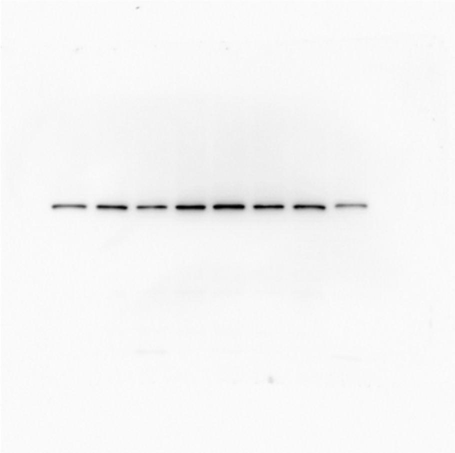

Full-length blot of Figure 3 (F) in the main text (pPKM2).

Full-length blot of Figure 3 (G) in the main text (PKM2).

Figure 5

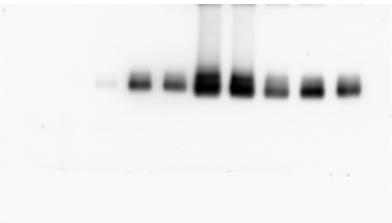

Full-length blot of Figure 5 (C) in the main text (Opsin, dark).

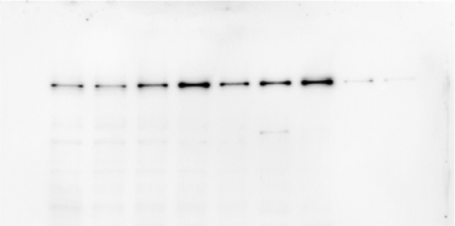

Full-length blot of Figure 5 (C) in the main text (α-3 Na/K ATPase, dark).

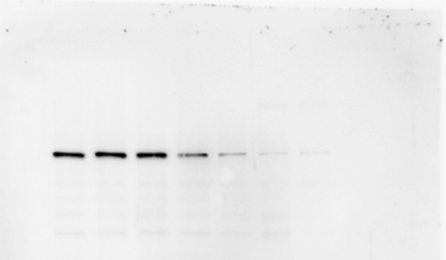

Full-length blot of Figure 5 (C) in the main text (pPKM2, dark).

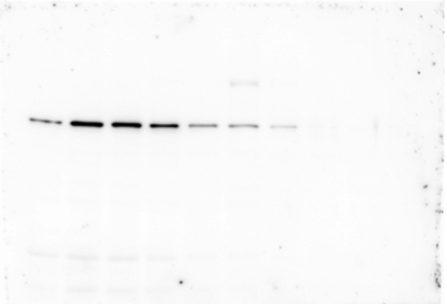

Full-length blot of Figure 5 (C) in the main text (PKM2, dark).

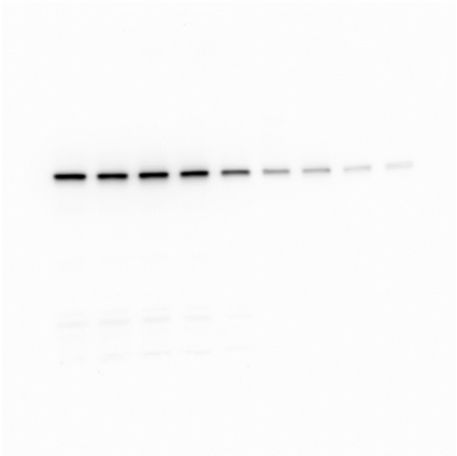

Full-length blot of Figure 5 (C) in the main text (PKM1, dark).

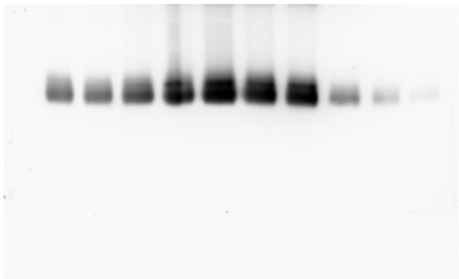

Full-length blot of Figure 5 (C) in the main text (Opsin, light).

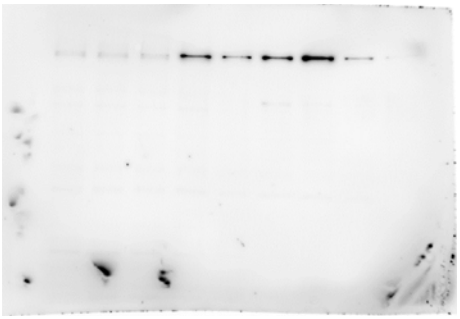

Full-length blot of Figure 5 (C) in the main text (α-3 Na/K ATPase light).

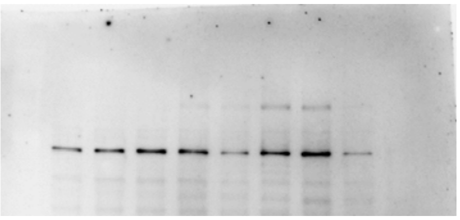

Full-length blot of Figure 5 (C) in the main text (pPKM2, light).

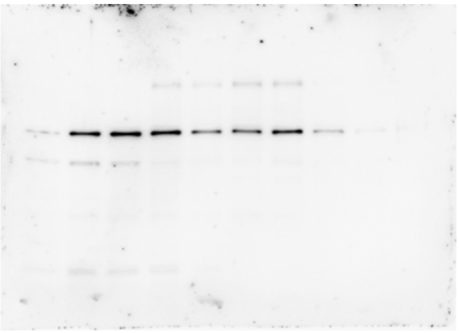

Full-length blot of Figure 5 (C) in the main text (PKM2, light).

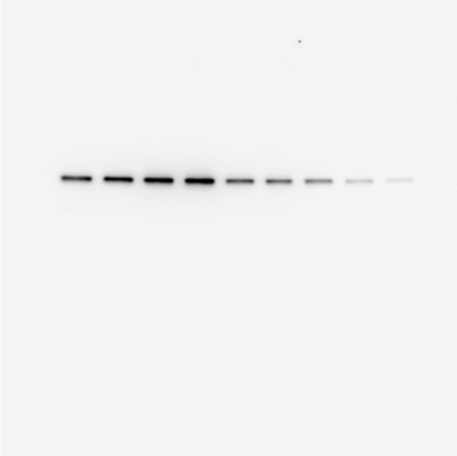

Full-length blot of Figure 5 (C) in the main text (PKM1, light).

Figure 6

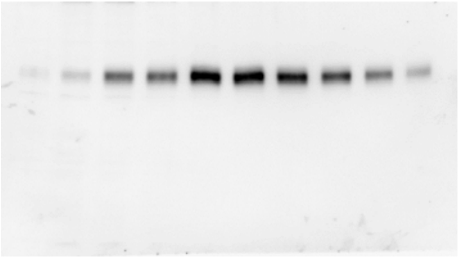

Full-length blot of Figure 6 (B) in the main text (M-opsin).

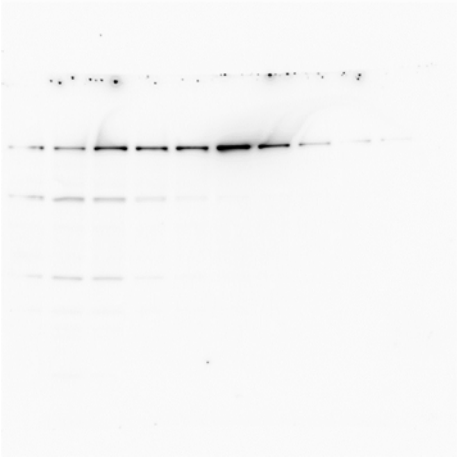

Full-length blot of Figure 6 (B) in the main text (α-3 Na/K ATPase).

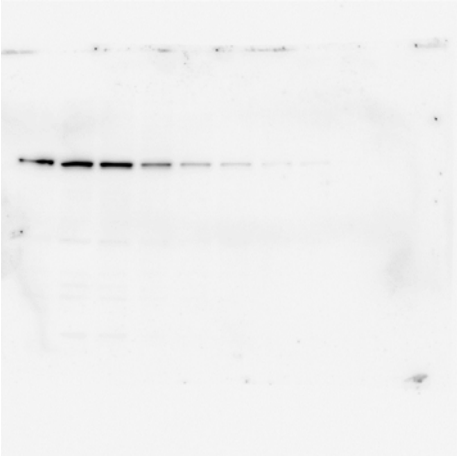

Full-length blot of Figure 6 (B) in the main text (pPKM2).

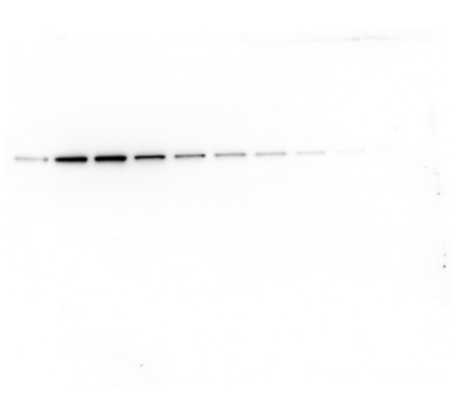

Full-length blot of Figure 6 (B) in the main text (PKM2).

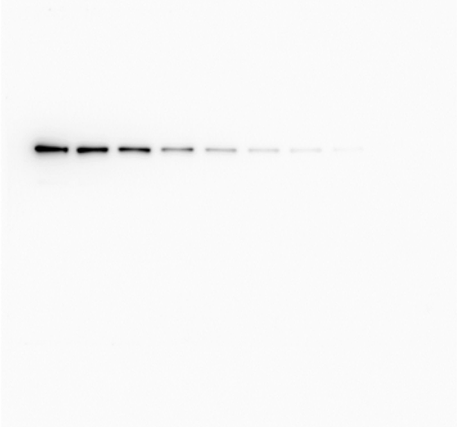

Full-length blot of Figure 6 (B) in the main text (PKM1).

Figure 7

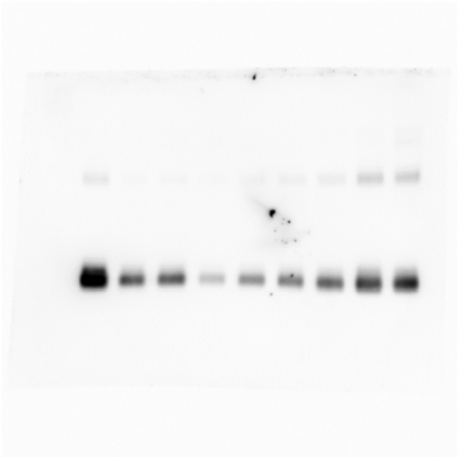

Full-length blot of Figure 7 in the main text (Opsin, dark).

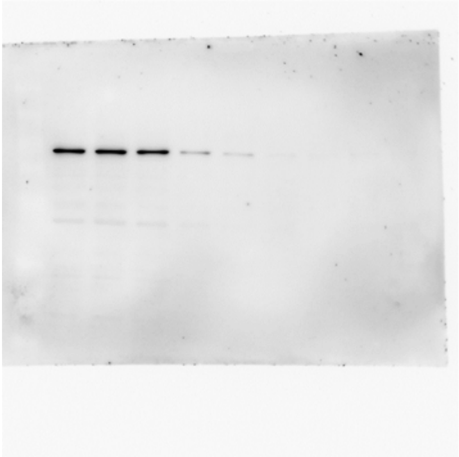

Full-length blot of Figure 7 in the main text (pPKM2, dark).

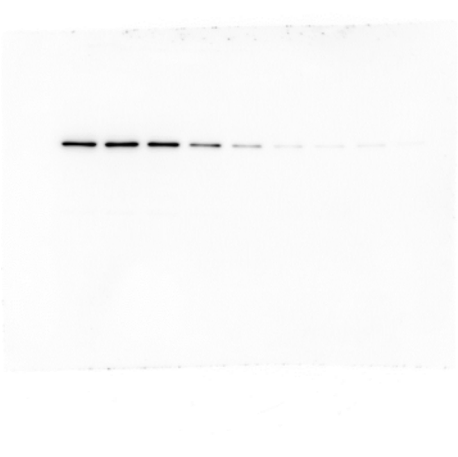

Full-length blot of Figure 7 in the main text (PKM2, dark).

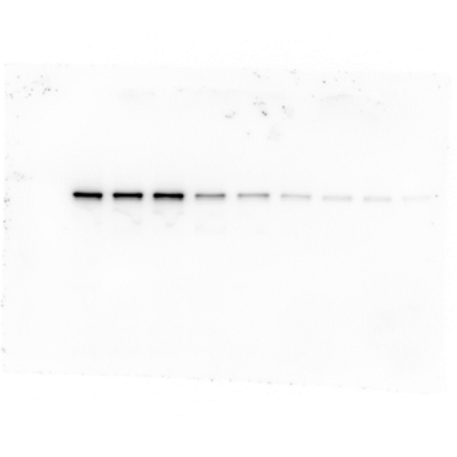

Full-length blot of Figure 7 in the main text (PKM1, dark).

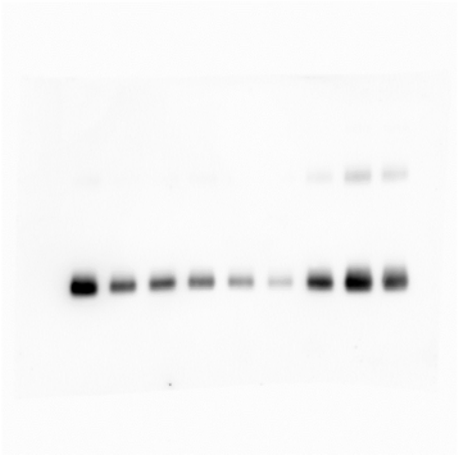

Full-length blot of Figure 7 in the main text (Opsin, light).

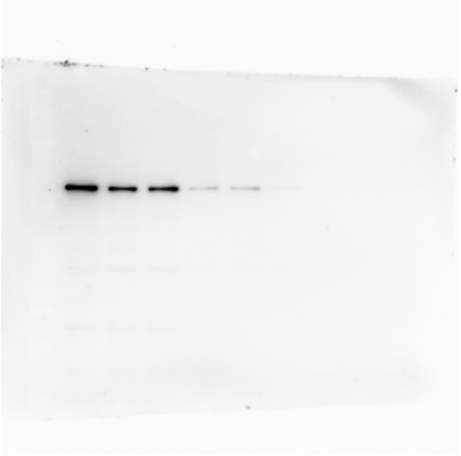

Full-length blot of Figure 7 in the main text (pPKM2, light).

Full-length blot of Figure 7 in the main text (PKM2, light).

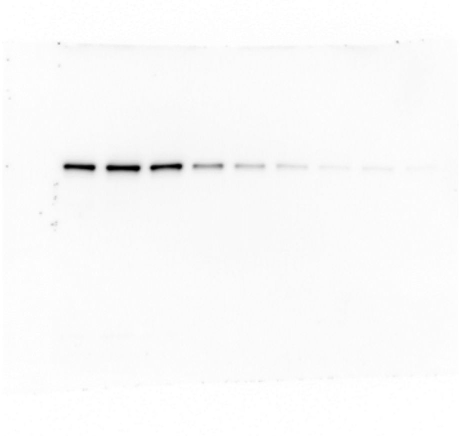

Full-length blot of Figure 7 in the main text (PKM1, light).

Figure 8

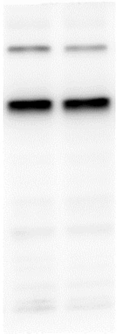

Full-length blot of Figure 8 (B) in the main text (pAkt-Light + DMSO).

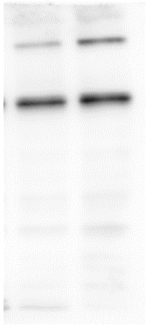

Full-length blot of Figure 8 (B) in the main text (pAkt-Light + LY294002).

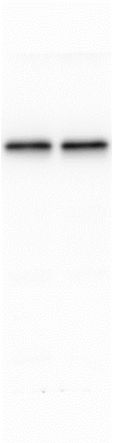

Full-length blot of Figure 8 (B) in the main text (Akt-Light + DMSO).

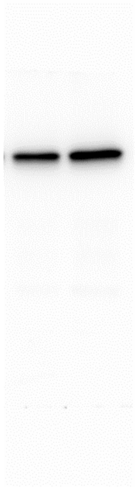

Full-length blot of Figure 8 (B) in the main text (Akt-Light + LY294002).

Full-length blot of Figure 8 (B) in the main text (pPKM2-Light + DMSO).

Full-length blot of Figure 8 (B) in the main text (pPKM2-Light + LY294002).

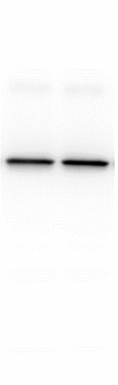

Full-length blot of Figure 8 (B) in the main text (PKM2-Light + DMSO).

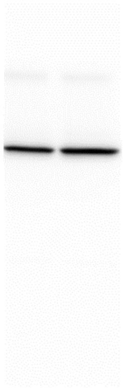

Full-length blot of Figure 8 (B) in the main text (PKM2-Light + LY294002)
